# ANA-Negative Hydralazine-Induced Pericardial Effusion

**DOI:** 10.1155/2017/3521541

**Published:** 2017-12-17

**Authors:** Vicken Zeitjian, Azar Mehdizadeh

**Affiliations:** ^1^Department of Internal Medicine, Maricopa Integrated Health System, Phoenix, AZ, USA; ^2^Department of Cardiology, Maricopa Integrated Health System, Phoenix, AZ, USA

## Abstract

This case describes a patient with pericardial effusion as a phenomenon of the drug-induced lupus erythematosus (DILE) syndrome due to hydralazine. The relevance of this case report lies in the fact that although hydralazine has been a known causative agent of DILE, its presentation may involve a negative anti-nuclear antibody (ANA) study. Pericardial effusion is a documented adverse effect as a result of hydralazine use. It is typically common to screen for DILE with the serum ANA test prior to proceeding to more costly and specific tests (i.e., anti-histone antibody). As per our literature review, this is the second case of hydralazine causing DILE with a negative ANA. As in our case, although the screening serum ANA is the initial next best step for suspicion of DILE by hydralazine, it is important to consider the diagnosis without ANA positivity.

## 1. Introduction

Hydralazine is a medication known to cause DILE, and case reports have revealed this since its first use in the United States in 1953. The DILE syndrome caused by hydralazine occurs in 5–10% of patients and commonly includes arthralgia, myalgia, fever, and serositis [1]. One of its more infrequent presentations reported in medical literature is pericardial effusion reported at <5% [2]. As per our literature review, this is the second case of hydralazine causing DILE with a negative ANA [3].

## 2. Case Report

A 21-year-old man with a history of ESRD secondary to FSGS, placement of a temporary right subclavian catheter, and renovascular hypertension presented to the emergency department with fevers and myalgia. The patient had been taking several blood pressure medications including hydralazine at a dose of 50 mg TID. Hydralazine was initiated 2 months prior to presentation. Review of systems was positive for fevers, chills, night sweats, myalgia, and pleuritic chest pain. He denied chest pain at rest, shortness of breath, nausea, vomiting, and diarrhea.

Initial vitals were remarkable for a temperature of 38.9°C, heart rate of 108, and blood pressure of 132/80. Physical exam was notable for a thin male with pleuritic chest pain on deep inspiration, slightly diminished heart sounds, no jugular venous distention, and a left brachiocephalic fistula with a palpable thrill.

**Figure 1:**
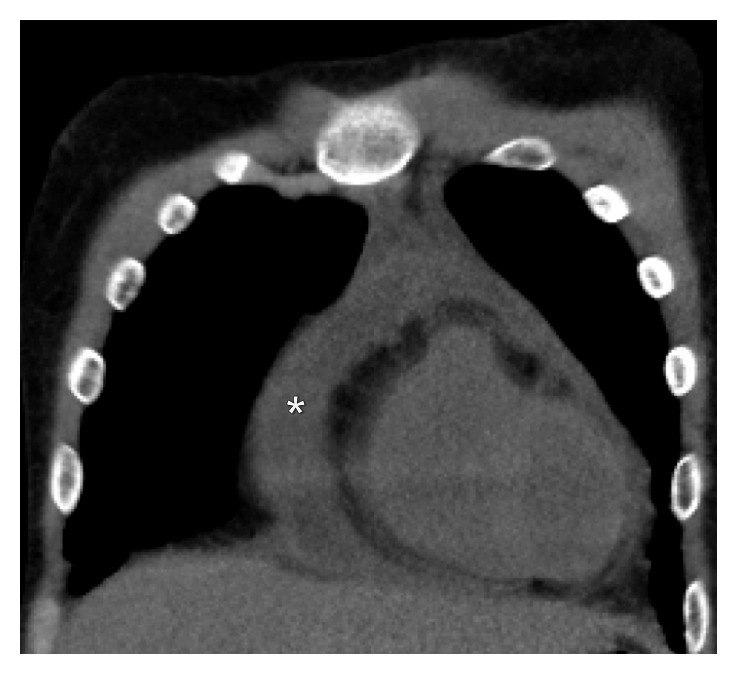
CT chest coronal view (pericardial effusion denoted by ∗).

**Figure 2:**
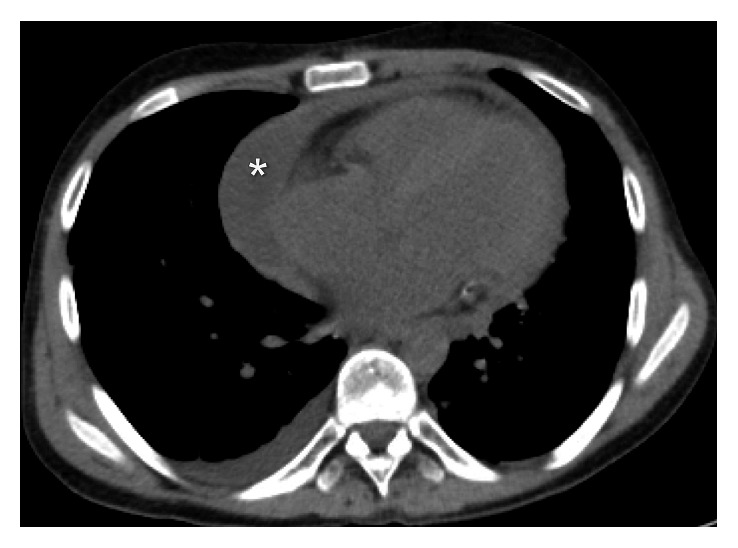
CT chest transverse view (pericardial effusion denoted by ∗).

**Figure 3:**
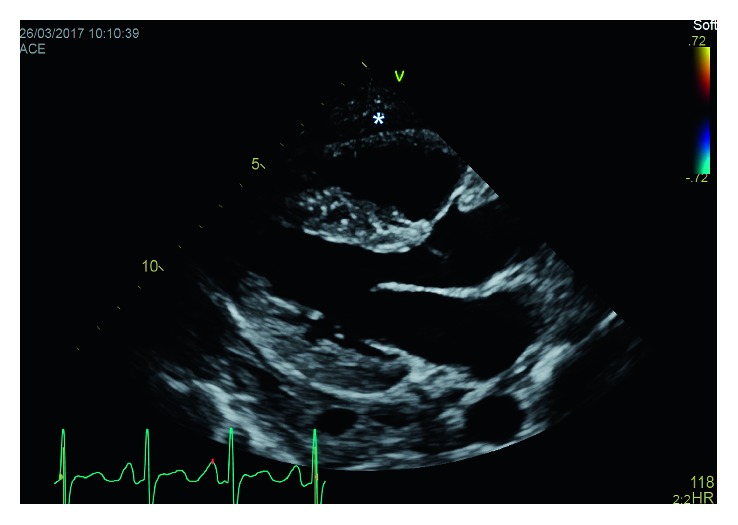
Transthoracic echocardiogram parasternal long-axis view (pericardial effusion denoted by ∗).

**Figure 4:**
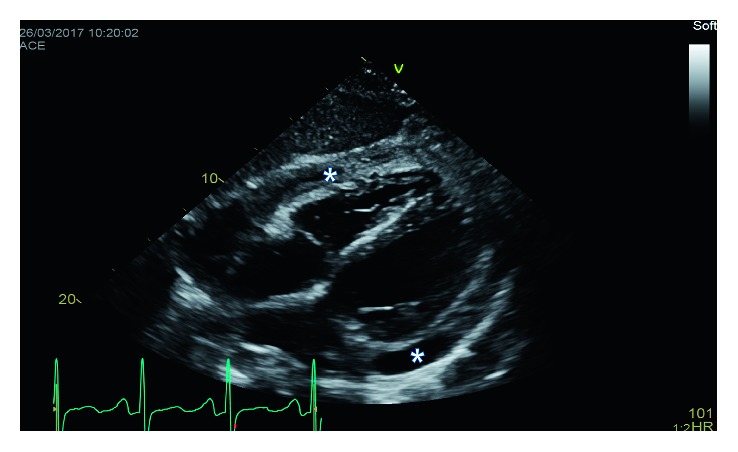
Transthoracic echocardiogram subcostal view (pericardial effusion denoted by ∗).

An electrocardiogram (EKG) was done which showed low voltage and was otherwise insignificant. A chest X-ray (CXR) showed an enlarged cardiac silhouette. Initial labs indicated anemia of chronic disease and ESRD but were otherwise unremarkable. Blood cultures were negative. A noncontrast CT chest was done which showed a moderate-sized pericardial effusion (Figures 1 and 2). An echocardiogram was done which identified a moderate-sized pericardial effusion without tamponade, no intracardiac mass, and presence of pericardial thickening (Figures 3 and 4). The pericardial effusion was suspected to be related to ESRD; however, 3 sessions of dialysis did not resolve the effusion.

Careful review of the medication list also brought forward the possibility of hydralazine-induced pericardial effusion as a presentation of DILE. ANA was ordered and found to be negative. Other etiologies of pericardial effusion were explored including pericarditis, autoimmune disease, malignancy, and myxedema; however, history and diagnostic and laboratory evaluations were not consistent with any of these disease processes.

Given the acuity of this disease process, there was still strong suspicion for drug-induced pericardial effusion. Anti-histone antibodies were ordered and were found to be positive. The diagnosis of DILE due to hydralazine causing pericardial effusion was made. The patient underwent pericardial drainage and pericardiectomy. Pericardial fluid cell count was significant for red blood cells of >700 k, as expected in this disease [2]. Pathologic analysis of the pericardium was consistent with fibrinous pericarditis. Cytology and pathology were negative for malignant cells. Infectious etiologies were ruled out, and prednisone 20 mg daily was started. Hydralazine was discontinued indefinitely, and patient's symptoms completely resolved.

## 3. Discussion

This case illustrates the importance of clinical suspicion as opposed to serologic markers as an approach to diagnosis. Most cases describing a DILE syndrome by hydralazine entail a positive ANA, and while negative ANA has been reported, it is a rare manifestation. In our case, although the screening serum ANA is the initial next best step for suspicion of hydralazine-induced lupus, it is important to consider the diagnosis without ANA positivity. Tests such as anti-histone antibody should be considered in the presence of strong clinical suspicion. Anti-chromatin (histone DNA macromolecule) antibodies have also been reported to be positive in up to 100% of people with this syndrome, although reports of using this test are scarce [4]. ANA and AHA are present in >95% of cases of this syndrome; however, further studies would be needed to identify what percentage of DILE have a positive AHA and a negative ANA [5].

To this day, there are over 70 medications implicated as possible etiologic agents of DILE [6]. Other commonly used medications aside from hydralazine include sulfamethoxazole, isoniazid, procainamide, phenytoin, methyldopa, chlorpromazine, infliximab, etanercept, and ACE inhibitors [7]. The list of medications reported to have caused an AHA positive and ANA negative are much smaller. Thus far, medications reported aside from hydralazine include lisinopril, quinidine, and minocycline [7–9]. Over 140 cases of DILE due to hydralazine have been reported; however, this is the second reported case with a negative ANA and the first presented by Alarcon-Segovia et al. [10].

Following the publication of the A-HeFT trial by Taylor et al. published in the NEJM in 2004, there has been an increase in the amount of hydralazine prescribed in patients with heart failure [2, 11]. For this reason, if pericardial effusion is suspected in patients with heart failure on combination hydralazine and isosorbide dinitrate, DILE should be in the differential. It was believed that DILE due to hydralazine was dose dependent; however, case reports now indicate that patients on lower doses of hydralazine (<100 mg) are not risk-free of this disease [5, 12].

With the increase of medications reported to cause DILE, it is important to keep in mind this potential side effect. In cases where suspicion is high, checking the AHA despite negative ANA may be indicated to identify the etiology of the disease. Anti-chromatin antibodies may also prove to be useful in diagnosis; however, given its limited medical literature, further studies would be needed to confirm its utility [4]. Steroids may be beneficial in the short term; however, discontinuing the offending agent is ultimately what will resolve this syndrome.

## 4. Conclusion

Hydralazine is a known cause of pericardial effusion as part of the DILE syndrome; however, the diagnosis should not be precluded in the setting of negative ANA if suspicion is high. Although rare, positive anti-histone antibody with a negative ANA is a diagnostic possibility for DILE as shown by this case report.
